# Developing a self-determination theory–informed coaching framework for the transition to residency

**DOI:** 10.5116/ijme.69af.f143

**Published:** 2026-03-27

**Authors:** Margaret Wolff, Matthew Kelleher, Luan Lawson, Noriko Anderson, William B. Cutrer, Benjamin Kinnear, Cindy Lai, Chemen Neal, Archana Pradhan, Maya Sardesai, Sally Santen, Jean Klig

**Affiliations:** 1 Department of Emergency Medicine and Pediatrics, University of Michigan Medical School, USA; 2 Departments of Pediatrics and Internal Medicine, University of Cincinnati College of Medicine, USA; 3 Medical Education and Student Affairs, Virginia Commonwealth University School of Medicine, USA; 4 Department of Neurology, University of California, San Francisco, USA; 5 Undergraduate Medical Education and Professor of Pediatrics, Vanderbilt University School of Medicine, USA; 6 Department of Pediatrics and Internal Medicine, University of Cincinnati College of Medicine, USA; 7 Department of Medicine, University of California, San Francisco School of Medicine, San Francisco, California, USA; 8 Obstetrics and Gynecology, Indiana University School of Medicine, USA; 9 Department of Obstetrics, Gynecology, and Reproductive Sciences, Robert Wood Johnson School of Medicine, USA; 10 Otolaryngology, Head and Neck Surgery, University of Washington School of Medicine, USA; 11 Emergency Medicine and Medical Education, University of Cincinnati College of Medicine, USA; 12 Emergency Medicine and Pediatrics, Harvard Medical School, Boston, Massachusetts, USA

**Keywords:** Coaching, medical education, self-determination theory, professional identity formation, Delphi method

## Abstract

**Objectives:**

To develop and content-validate a consensus-based, Self-Determination Theory (SDT)–informed single-session coaching framework for the transition from medical school to residency (TTR).

**Methods:**

We conducted a modified Delphi study with 13 medical educators from 11 U.S. medical schools, purposively sampled for diversity of geography, institutional type, and coaching infrastructure. Eligibility required experience coaching in medical education. Using a nominal group technique, panelists generated 125 prompts and 12 skills across the coaching arc (opening, exploring, planning, closing). Duplicates were consolidated by consensus rules. In Round 1, panelists rated items on a 1–10 scale; consensus was >80% rating 9–10. In Rounds 2–3, binary yes/no voting determined inclusion or exclusion. Quantitative analysis included medians, interquartile ranges, and Kendall’s coefficient of concordance (W) for Round 1. Qualitative comments were analyzed with rapid content analysis to guide revisions.

**Results:**

All panelists completed each round (100% retention). In Round 1, 17 questions and 2 skills met consensus (median = 9, IQR 0–1; W = 0.78). Round 2 retained 2 questions and 1 skill, and excluded 62 items. Round 3 added 6 questions and 1 skill, yielding a final framework of 25 prompts and 4 skills. Prompts were distributed across phases (opening 5, exploring 9, planning 7, closing 4) and mapped to SDT needs (autonomy 9, competence 8, relatedness 8). Panelists affirmed clarity, feasibility, and acceptability for non-coach faculty.

**Conclusions:**

This consensus-derived framework provides a pragmatic, SDT-grounded tool for coaching at the TTR. Future studies should evaluate feasibility, fidelity, and learner outcomes.

## Introduction

Coaching has emerged as a powerful tool in medical education, supporting learners in developing self-awareness, setting goals, and navigating the complexities of their professional journeys. ^1^^-^^6^

When delivered effectively, coaching can enhance professional identity formation, foster adaptive learning strategies, and promote a growth mindset.^1^^,^^3^^,^^7^ Yet, despite these benefits, access to coaching remains limited across undergraduate medical education (UME) programs.^8^ Longitudinal coaching relationships, while ideal, require sustained institutional investment, trained personnel, and structural support, all of which may be unavailable in resource-constrained environments.^9^^-^^11^

Self-Determination Theory (SDT), a framework for understanding human motivation, forms the theoretical basis for coaching practices aimed at supporting learners’ basic psychological needs of autonomy, competence, and relatedness.^12^ These needs are essential for fostering intrinsic motivation, well-being, and professional growth. ^12^^-^^14^

Coaching conversations, particularly those grounded in reflection and goal setting, can foster autonomy by helping learners articulate personal aspirations; competence by highlighting strengths and planning for skill development; and relatedness through supportive dialogue with a trusted educator. These needs are especially salient during the transition to residency (TTR), a period marked by heightened uncertainty and vulnerability, making it an ideal target for interventions that support motivation, identity, and self-efficacy.^6^^,^^15^

Although several longitudinal coaching models exist, there is currently no consensus-based, SDT-informed single-session coaching framework designed specifically for the TTR. This represents a critical knowledge gap, as most institutions lack the resources to implement sustained coaching programs. Our aim was to develop a consensus-based, SDT-informed single-session coaching conversation framework for the TTR and to evaluate its acceptability and content validity. The intended users are faculty across specialties, including those without formal coaching training, who support learners at this transition. We intentionally focused on a single-session framework to maximize accessibility, scalability, and generalizability.

## Methods

We utilized a modified Delphi technique to establish consensus on the structure and core questions of a coaching conversation for learners transitioning to residency.^16^^,^^17^ The modified Delphi technique is an iterative approach that gathers and refines expert input over successive rounds to achieve consensus. A team member (MW) with extensive experience in the modified Delphi method guided the methodology and facilitated the process but did not participate in voting.

We convened a multi-institutional panel of 13 medical educators with expertise in medical education coaching. Eligibility criteria included prior experience coaching undergraduate medical education (UME) or graduate medical education (GME) learners or designing institutional coaching programs. Individuals without coaching experience were excluded. Panelists were recruited through national coaching networks to ensure diversity in institutional type, geography, and coaching infrastructure. Six represented schools with formal coaching program, while others came from institutions with more limited resources. The final sample size (n=13) aligns with recommended Delphi panels of 10-18 experts to ensure robust consensus.^16^ All panelists participated in every round (100% retention).

### Recruitment and eligibility

Panelists were invited by email, with disclosures of potential conflicts of interest requested and reviewed. No conflicts were identified. All participants consented to voluntary participation. Learners were not included, as the primary purpose was expert-derived content validation rather than end-user testing.

We initiated the consensus-building process with a nominal group technique, a structured method designed to promote broad participation while minimizing dominance effects. Panelists first reviewed the four phases of the coaching arc (opening, exploring, planning, closing) and independently generated 5–10 potential coaching questions for each phase, along with essential coaching skills. ^18^This approach yielded a broad and diverse pool of questions that reflected the varied experiences of panelists. Duplicates and overlapping submissions were consolidated by the research team using consensus rules, with all original wording preserved in the event of re-introduction during later rounds. Through this process, 125 initial prompts and 12 skills were identified and used to construct the Round 1 Delphi survey.

Responses were compiled into a Qualtrics survey. In Round 1, panelists rated each item from 1 (“do not include”) to 10 (“definitely include”). Items with >80% of ratings 9-10 were retained; others moved forward.^17^ A comment box allowed panelists to provide qualitative feedback and suggested revisions. Qualitative comments were coded using a rapid content analysis approach which allowed the research team to synthesize feedback into domains such as wording clarity, redundancy, and alignment with SDT for integration into subsequent rounds.^17^^,^^19^ This method provides a systematic yet efficient process for incorporating qualitative data into iterative consensus methods.^19^  Content was then integrated before subsequent rounds.

A second Zoom meeting allowed discussion of Round 1 results and wording clarifications. In Round 2, panelists voted “yes” or “no” for inclusion of remaining items. Items with > 80% yes were retained, and items with >80% no were removed. This shift from numeric rating to binary voting was prespecified to facilitate efficient narrowing after the first round.

In Round 3, unresolved items were re-discussed. Items reaching consensus were retained or excluded accordingly. In Round 4 (consensus meeting), the final framework was presented for review and minor adjustments; no survey ratings were collected. Throughout all meetings, individual survey data were de-identified before discussion to minimize dominance bias, and MW facilitated discussions without contributing content.

### Consensus criteria and stopping rules

Consensus was defined as >80% agreement, consistent with Delphi standards.^17^ Near-threshold items (>70% but <80%) were re-discussed until disposition was achieved by majority vote. Stopping rules were prespecified as three Delphi rounds or earlier if stability was reached.

### Data analysis

Quantitative analysis of Round 1 ratings included means, medians, and interquartile ranges; Kendall’s coefficient of concordance (W) was calculated to estimate within-round agreement among panelists. Subsequent rounds were summarized descriptively by percent agreement relative to the a priori ≥80% threshold. Qualitative comments from each round were coded using a rapid content analysis approach and used to iteratively refine items between rounds.

This study was reviewed by the Institutional Review Board and was determined to be exempt from further review. The exemption was granted under Category 1 of the federal exemption criteria at 45 CFR 46.104(d), which applies to research conducted in established or commonly accepted educational settings involving normal educational practices that are not likely to adversely impact students’ opportunity to learn or the assessment of educators. In this study, participants were expert educators evaluating educational content; no learner or patient data were collected. Participation was voluntary, responses were confidential, and no incentives were provided.

## Results

**Figure 1:**
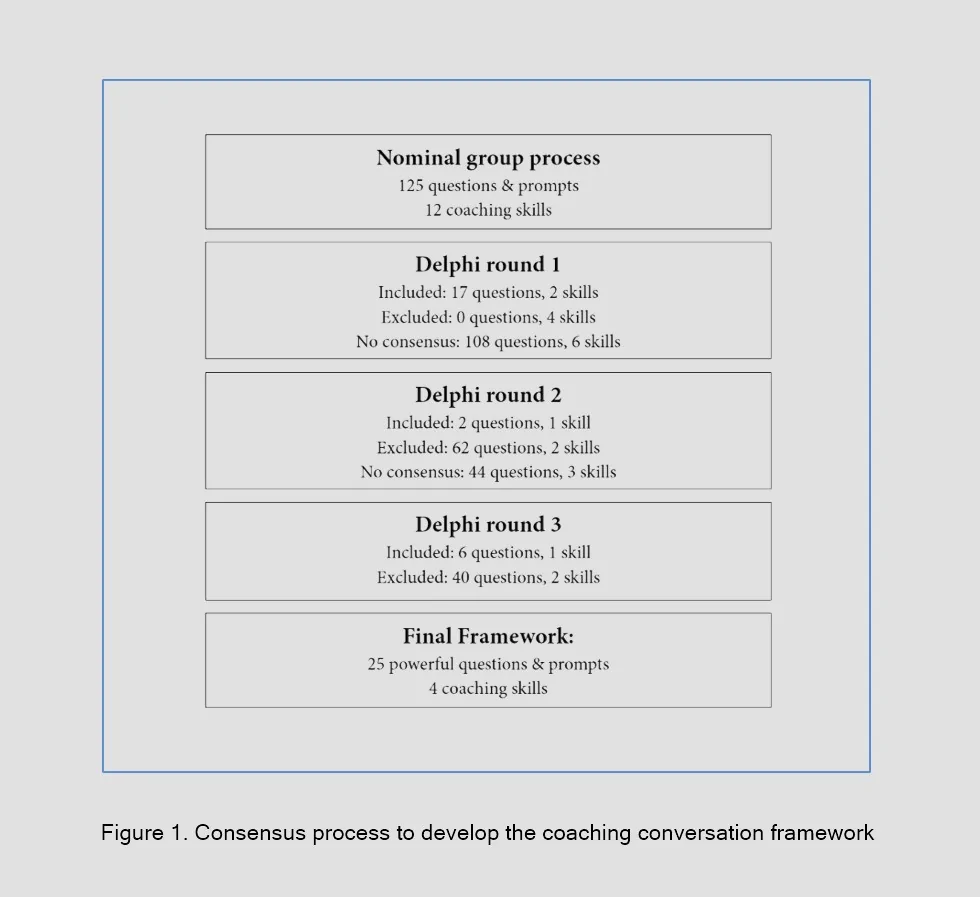
Consensus process to develop the coaching conversation framework

The Delphi panel identified a set of powerful coaching questions and essential skills to support structured coaching conversations (Figure 1). All 13 panelists participated in each survey and meeting, with 100% retention across all rounds.

The initial nominal group process generated 125 questions distributed across the four stages of the coaching framework: 13 questions in the “opening the session” phase, 47 in the “exploring the topic and eliciting reflection” phase, 49 in the “exploring options and developing an action plan” phase, and 16 in the “closing” phase. Panelists also proposed 12 essential coaching skills. After deduplication and merging of overlapping submissions, the Round 1 survey contained 125 unique questions and 12 skills.

After Round 1, 17 questions and 2 skills reached high agreement (median = 9, IQR 0-1; >80% rating 9-10). In Round 1, Kendall’s coefficient of concordance (W) was calculated to assess the degree of agreement among panelists’ 1–10 ratings. The value of W = 0.78 indicated strong concordance, reflecting high stability in expert ratings at this stage. No items were excluded at this stage, and 108 questions with 10 skills were advanced to the next round.

In Round 2, panelists used yes/no voting to evaluate the remaining items. Two additional questions and one skill achieved >80% inclusion, while 62 questions and 2 skills were excluded.  Forty-four questions and seven skills progressed to Round 3. In this final rating round, six more questions and one additional skill reached consensus. Forty questions and two skills were excluded. Because Rounds two and three used binary yes/no voting rather than ranked data, stability was assessed using the proportion of panelists voting for inclusion or exclusion, consistent with Delphi methodology.

Overall, the process narrowed the list from 125 questions and 12 skills to a final framework of 25 powerful question prompts and 4 essential coaching skills. Skills were retained based on frequency of endorsement and consensus discussions emphasizing practicality and alignment with core coaching principles, including active listening, growth mindset, preparation, and minimizing distractions.

Qualitative feedback highlighted the importance of clarity, SDT alignment, and feasibility for non-coach faculty. Revisions included simplifying wording, removing redundancy, and ensuring prompts encouraged autonomy, competence, and relatedness. Of the final 25 questions, 9 primarily supported autonomy, 8 competence, and 8 relatedness, directly mapping to SDT domains.

These elements formed the final coaching conversation framework (Table 1), representing a strong consensus on practical strategies that faculty can use to guide effective coaching conversations during the transition to residency.

## Discussion

In this study, we used a structured nominal group technique followed by a modified Delphi process to develop a structured coaching framework to support learners during the transition to residency (TTR). From an initial pool of 125 questions and 12 skills, the panel distilled a final framework of 25 questions and 4 essential coaching skills. These prompts are designed to guide reflection, self-assessment, and action planning across the natural arc of a coaching conversation.

Importantly, this study addresses a specific gap: although longitudinal coaching models are well described, no consensus-derived, SDT-informed single-session framework for the TTR has previously been developed. Our results demonstrate the feasibility of reaching strong expert consensus on such a framework, offering a pragmatic tool for use across diverse educational settings.

The final framework offers an accessible entry point for educators, even those without formal coaching training, to engage learners in meaningful, future-focused conversations. Its adaptability and low resource requirements make it especially relevant for institutions unable to support longitudinal coaching programs.

Self-Determination Theory (SDT), a well-established theory of human motivation, provides the theoretical underpinning for our study by explaining the value of coaching to support learners’ basic psychological needs for autonomy, competence, and relatedness—key drivers of intrinsic motivation, well-being, and professional development.^12^  Coaching interactions grounded in reflective dialogue and goal-setting naturally promote autonomy by centering the learner’s personal values and aspirations, competence by helping learners recognize their strengths and make actionable plans, and relatedness by fostering supportive, learner-centered relationships. These psychological needs become particularly salient during transitions such as the shift from medical school to residency, when learners often experience increased vulnerability, uncertainty, and self-doubt. By mapping prompts to the basic psychological needs described in SDT, the framework is theoretically grounded in motivation science and designed to support learner engagement and professional identity formation during a critical phase of training.

The TTR is widely recognized as a period of heightened stress and uncertainty, with national bodies such as the Undergraduate Medical Education - Graduate Medical Education Review Committee (UGRC) highlighting the importance of coaching during this transition.^6^ Our findings align with these recommendations by providing a structured, consensus-derived approach that institutions can adopt. Because transitions occur throughout medical training, the framework may also be useful beyond TTR. By emphasizing exploration of uncertainty, surfacing internal barriers to growth, and building actionable plans, the frameworksupports conversations that normalize struggle, counter imposter syndrome, and encourage reflection on strengths.

Such dialogue fosters a growth mindset and professional identity formation, both of which are essential during critical career transitions. The final months of medical school offer a rare window for intentional reflection, and structured coaching conversations at this stage may enhance readiness for residency while supporting learners’ long-term professional development.

This study has several limitations. First, the panel was small (n=13) and limited to expert educators, so learner perspectives were not included. Second, although we calculated Kendall’s coefficient of concordance (W) to demonstrate strong agreement in Round 1, subsequent rounds used binary voting, which did not permit calculation of stability statistics. Agreement in those rounds was therefore assessed by proportion of inclusion or exclusion, which may provide a less nuanced assessment of consensus. Third, consensus rules shifted between rounds, from a 1–10 scale to yes/no voting, to streamline the process. While prespecified, this change may have influenced outcomes. Fourth, although anonymity was maintained for survey ratings, live Zoom discussions may have introduced potential for dominance bias despite structured facilitation and safeguards. In addition, the reduction of coaching skills from 12 to 4 was based on panel discussion and endorsement frequency rather than formal psychometric analysis. Finally, the framework has not yet been tested in real-world coaching sessions, so its acceptability and effectiveness for learners remain unproven.

These limitations temper the claims that can be made. Rather than a definitive model, the framework should be viewed as a pragmatic starting point that requires local adaptation and brief faculty development.

**Table 1:** The Coaching Framework – A 5 Step Approach: Build. Explore. Reflect. Plan. Close

	Key Coaching Skills
	Prepare in advance
	Minimize distractions
	Use active listening
	Embrace a growth mindset
	Build the relationship & set the agenda
	Provide an overview of the session
	Identify specific area of focus
	Ask the learner to identify ideal outcome of conversation
	A firm safe space and emphasize discussion won’t be shared
	Explore the focus
	Normalize their uncertainty
	Help them explore their uncertainty:
	Tell me more about (use specific area of focus)
	Help me understand more…
	What emotions does this evoke for you?
	What are you excited about/nervous about related to this transition?
	What is the impact of this on you?
	Reflect on prior experiences to identify strengths & skills that may help with the transition
	Think of a major transition you’ve been through in the past. How did you navigate that?
	What did you learn about yourself from that experience?
	What strengths or skills helped you navigate this?
	What are your opportunities for growth in this upcoming transition?
	How will embracing this next challenge help you?
	What is your new way of seeing this?
	Define success & create an action plan using WOOP^20^
	Wish - What is one wish you would like to fulfill in this transition? Pick a wish that feels challenging, but that you can reasonably fulfill.
	Outcome - What would be the best outcome if your wish comes true? How would you feel?
	Obstacle - What within you holds you back from fulfilling your wish? What might stop you? It might be an emotion, an irrational belief, or a bad habit. Identify your main inner obstacle.
	Plan - What can you do to navigate your obstacle? Identify one effective action you can take or one effective thought you can think to overcome your obstacle. If this happens…I will do this…
	Close the conversation
	Signal wrap-up.
	Have learner identify how they will stay accountable.
	Have learner summarize action plan.
	Express gratitude for sharing.
	Give the learner the opportunity to have any last words to close the conversation.

Future research should focus on cognitive interviews with learners to assess clarity and acceptability of prompts, pilot feasibility and fidelity studies of faculty use, mapping of prompts to observed SDT outcomes such as self-efficacy and preparedness, and multi-site trials to evaluate generalizability. Additional work should also explore whether use of the framework supports long-term outcomes such as resilience, well-being, and professional identity formation.

Despite these limitations, this study contributes a practical and scalable framework for coaching at the transition to residency. By broadening access to high-quality, SDT-informed coaching conversations at a pivotal moment in training, this framework may help learners navigate uncertainty, strengthen self-awareness, and prepare more confidently for the demands of early residency.

## Conclusions

We developed a practical, consensus-based coaching conversation framework to support learners during the transition from medical school to residency. Designed for ease of implementation, this framework offers institutions a scalable, low-resource approach to facilitating reflective conversations that foster self-awareness, goal-setting, and professional growth. Grounded in SDT, the framework supports learners’ psychological needs for autonomy, competence, and relatedness. While promising, the framework should be viewed as a pragmatic starting point that requires local adaptation and further study. Future work is needed to examine its feasibility, fidelity, and impact on learner outcomes. By broadening access to coaching at a pivotal moment in training, this framework may help enhance learner readiness and well-being during one of the most critical transitions in medical education.

### Acknowledgments

Funding: American Medical Association ChangeMedEd Initiative.

### Conflict of Interest

The authors declare that there is no conflict of interest.
